# mgikit: demultiplexing toolkit for MGI fastq files

**DOI:** 10.1093/bioinformatics/btae554

**Published:** 2024-09-11

**Authors:** Ziad Al Bkhetan, Sen Wang

**Affiliations:** South Australian Genomics Centre, SAHMRI, Adelaide, SA, 5001, Australia; Australian BioCommons, The University of Melbourne, Melbourne, VIC, 3010, Australia; South Australian Genomics Centre, SAHMRI, Adelaide, SA, 5001, Australia

## Abstract

**Summary:**

MGI sequencing is reported to be an inexpensive solution to obtain genomics information. There is a growing need for software and tools to analyse MGI’s outputs efficiently. *mgikit* is a tool collection to demultiplex MGI fastq data, reformat it effectively and produce visual quality reports. mgikit overcomes several limitations of the standard MGI demultiplexer. It is highly customizable to suit different kinds of datasets and is designed to achieve high performance and optimal memory utilization.

**Availability and implementation:**

The tool and its documentation are available at: https://sagc-bioinformatics.github.io/mgikit/.

## 1 Introduction

MGI Tech launched a series of new NGS equipments based on DNBSEQ technology. These sequencers have been reported to have comparable accuracy to Illumina sequencers for different types of sequencing libraries (Jeon *et al.* 2021, Kim *et al.* 2021). However, they are more cost-effective and have high throughput reaching 6 TB of data a day, as per the case of the T7 sequencer. These reasons pave the way for MGI sequencers to be widely utilized in the genomics field and therefore encourage the development of software that can analyse such data.

**Figure 1.**
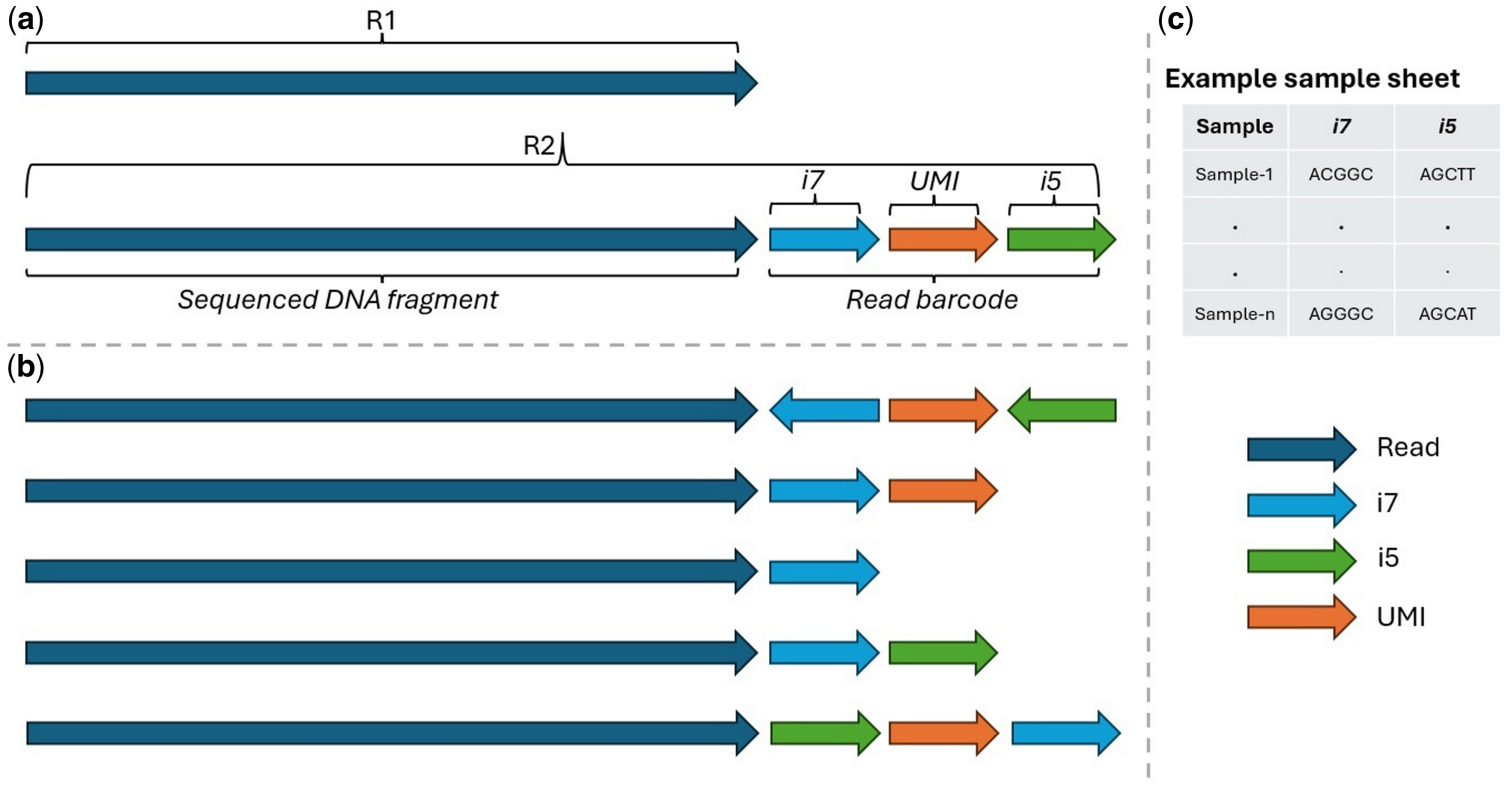
Examples of sequenced reads include indices and UMI. (a) Both reads R1 and R2, read barcode at the tail of R2 containing the dual index (i7 and i5) and an UMI. The sequenced regions located at these indices will be compared with the relevant expected indices from the sample sheet. If the sequences are matching, the read is assigned to the relevant sample. (b) Possible different locations and forms for the indices and UMI in the read barcode where the direction of the arrow represents if the read is in a reverse complementary form or not. They are according to their order: 1—Dual index in reverse complementary form and an UMI. 2—Single index (i7) and an UMI. 3—Single index alone. 4—Dual index without UMI. 5—Dual index and an UMI in a different order. (c) An example of a sample sheet containing sample ID and the sample dual index i7 and i5 sequences. During demultiplexing, these indices will be compared to the sequenced ones appearing in the read barcode at the tail of R2 shown in (a) and (b) to map the sequenced reads to the samples.

To optimize for cost and run time, sequencing facilities pool and sequence multiple samples or libraries simultaneously within a single run. During the library preparation, short unique DNA sequences (single index ‘i7’ or dual index ‘i7 and i5’) are attached to the DNA fragments of the same sample. In some cases, another short DNA sequence called UMI (Unique Molecule Identifier) is also attached to the DNA fragments to identify each molecule in a sample library. UMIs are used for error correction and accuracy improvement of the sequencing while single/dual index is used to map the sequenced reads to their origin sample. An example is illustrated in Fig. 1.

The process of comparing the sequenced indices with the original ones used during library preparation for read-sample mapping is called demultiplexing and is performed routinely by any sequencing facility before handing over the data to the users.

Due to sequencing and base calling errors, sequenced indices do not necessarily match perfectly with the expected ones. Many approaches were proposed to tackle this issue, including the official demultiplexers such as Illumina demultiplexers [bcl2fastq (https://sapac.support.illumina.com/sequencing/sequencing_software/bcl2fastq-conversion-software.html) and CASAVA (https://support.illumina.com/help/CASAVA_QRG_Help/Content/Vault/Informatics/Sequencing_Analysis/CASAVA/swSEQ_mCA_FrontPageQRG.htm)] and MGI demultiplexer [splitBarcode (https://github.com/MGI-tech-bioinformatics/splitBarcode)]. These demultiplexers are the standard tools used for Illumina and MGI datasets and are conservative and exact methods that look for perfect matches with some flexibility to allow a few base mismatches based on hamming distance and without considering quality scores. Other approaches are more sophisticated yet prone to errors such as statistical and machine learning approaches. These methods demultiplex the ambiguous reads by maximizing the likelihood of matching specific indices (Renaud *et al.* 2015, Yi *et al.* 2015).

The standard procedure by sequencing facilities is to use the official demultiplexers that are very accurate but might miss some reads. The remaining undetermined reads (i.e. reads that did not match with any sample during the demultiplexing) can be re-demultiplexed in a later stage by the users who have different preferences on what demultiplexer to use depending on their experience and the nature of their data.

MGI sequencers output large fastq files with different read headers and file naming than Illumina outputs. The end of the reverse read (R2) for paired-end or the forward read (R1) for single-end is the ‘read barcode’ that is not part of the actual DNA fragment that was sequenced and it only contains the sample’s indexes (i7 and i5) and unique molecule identifier (UMI) that were attached during the library preparation.

This format is not supported by available demultiplexers that are developed mainly for Illumina data and it can only be demultiplexed via MGI’s official tool ‘splitBarcode’. However, splitBarcode disregards the UMIs in the input data and does not write it into the output files which can be problematic for many analyses. Its output format (MGI format for file naming and read headers) is not compatible with some downstream analysis tools that expect Illumina format. In addition, it requires the user to know upfront where to find the indexes within the read’s barcode and it does not support demultiplexing mixed libraries within the same run in one execution. Although some of these challenges can be resolved by reformatting MGI output data into an acceptable format by some available demultiplexers, this solution is impractical in real scenarios as it requires doubling the storage for the reformatted data (up to 6 TB for one run from T7 sequencers) and will increase the total performance time significantly as reading/writing compressed text is very slow and this needs to be done twice (reformatting then demultiplexing). To our knowledge, there are no tools that reformat MGI fastq files, and it is more efficient to develop a tool that resolves all these issues in one execution rather than automating a workflow of multiple tools.

In this application note, we provide *mgikit*, a software kit, to demultiplex MGI fastq data, detect barcode templates and generate demultiplexing and quality reports that can be converted to HTML reports through *mgikit-multiqc* plugin (available at https://github.com/sagc-bioinformatics/mgikit-multiqc) that integrates with the MultiQC tool (Ewels *et al.* 2016). *mgikit* is written in the RUST programming language. A comprehensive documentation and user guide are available at the tool web pages https://sagc-bioinformatics.github.io/mgikit/.

## 2 Results

### 2.1 fastq demultiplexing

The ‘*demultiplex*’ command of *mgikit* takes input fastq files (single/paired-end), a sample sheet that contains a list of sample IDs and their indices (single or dual), and a ‘barcode template’ that represents the locations of the indices in the read barcode as described in the tool documentation. The tool extracts the sequenced indices from the reads and compares them to the sample indices from the sample sheet allowing specific hamming distance (base mismatches) that can be customized by the user. The read is assigned to the best matching sample, i.e. minimal hamming distance to its indices. If multiple samples match the same read with the same hamming distance, the read is reported as ambiguous, otherwise, the read is undermined as it does not match any sample. This approach does not consider quality scores in demultiplexing and is affected by base-calling errors, but tolerates a few errors by accepting matches with a hamming distance >0 (usually <2 errors per index). The same approach is used in the official demultiplexers by Illumina and MGI.

To achieve efficient index matching by allowing a specific mismatch threshold (*m*), *mgikit* generates all possible indices from the indices in the sample sheet allowing 0 to *m* mismatches and assigning these indices to the relevant samples. For example, for m=1 and one sample with a single index ‘ACG’, the tool will create the following indices (‘ACG’, ‘CCG’, ‘GCG’, ‘TCG’, ‘NCG’, ‘AAG’, ‘AGG’, ‘ATG’, ‘ANG’, ‘ACA’, ‘ACC’, ‘ACT’, ‘ACN’), any read that matches any of these indices will be reported for the same sample. The data structure can be constructed considering single or dual indices and it resolves any issue related to overlapping indices across multiple samples especially when *m* is large enough which is not recommended. This dictionary-based data structure achieves a quick and flexible search for matching indices. It requires one dictionary fetch for each index to find the associated sample regardless of the user-defined threshold for the allowed mismatches.

*mgikit* functionalities are highly optimized for performance time through buffering the compression and output data to minimize writing-to-disk operations. The main features provided by the *mgikit* demultiplexer compared to splitBarcode are highlighted in Table 1.

**Table 1.** Feature comparison between *mgikit* and splitBarcode.^a^

Feature	mgikit	splitBarcode
Single-read data support	Yes	No
Paired-end data support	Yes	No
10X fastq data support	Yes	No
Mixed libraries support	Yes	No
Index diversity support	Yes	No
Runs with UMI support	Yes	No
Demultiplexing reports	Text & HTML	Text
Data quality reports	Text & HTML	Text
Multi-threading	Yes	Yes
Output file naming	Illumina & MGI	MGI
Header format	Illumina & MGI	MGI

a Index diversity represents the case when the same sample has multiple indices in the run (multiple entries in the sample sheet for the same sample). In this case, mgikit outputs all the reads that match the different sample’s indices to the same files. mgikit’s HTML reports are generated through mgikit-multiqc plugin. Multi-threading is implemented in mgikit in all versions after v0.1.6.

### 2.2 Detection of barcode template

Sample indices and UMI are expected to be in the read barcode, but in some situations, the order of these short sequences in the read barcode is unknown upfront the demultiplexing or whether they exist in the read barcode as they appear in the sample sheet or in a reverse complementary form. Figure 1 demonstrates some possible scenarios for different locations and forms for the indices and UMI within the read barcode. The exact location of each index and the UMI is required by *mgikit* to extract the sequence within these regions and then compare it with the indices in the sample sheet to find the relevant sample. Detecting the location of these indices in the read barcode and whether they need to be converted into the reverse complementary form can be facilitated via the ‘*template*’ command. *mgikit* tries all possible combinations of the indexes and UMI considering the sequence and its reverse complementary form and then selects the template that achieves the maximum number of matches with the samples’ indexes. For example, if a run has dual index i7 (8 bp) and i5 (8 bp), the read barcode is expected to be 16 bp long (the sum of i7, i5 lengths). Potentially the sequenced i7 and i5 can be located in the first 8 bp, then i5 the other 8 bp or vice versa. The sequenced i7 and i5 can be matched with the indices in the sample sheet as they appear or with their reverse complementary form. *mgikit* will investigate all these options including having an UMI and report the best matching template (the one that leads to the maximum matches with the sample sheet). This utility is flexible and can detect diverse templates for mixed libraries.

### 2.3 Demultiplexing reports

*mgikit* reports information on demultiplexing and read quality for each run. It also merges reports from multiple lanes into a comprehensive report for the whole run as well as for samples from different projects within the same run. *mgikit* reports can be parsed by the *mgikit-multiqc* plugin, as explained in the online documentation (https://github.com/sagc-bioinformatics/mgikit-multiqc), to generate user-friendly html reports integrated into multiqc (Ewels *et al.* 2016) reports. *mgikit* provides ‘*report*’ functionality to merge multiple reports from multiple lanes together.

### 2.4 Accuracy and performance evaluation

*mgikit* is expected to perfectly assign all reads to the expected samples with respect to the predefined allowed mismatches. The tool successfully passes all accuracy validation tests on simulated datasets (available on the GitHub repository) for several configurations and scenarios with every change pushed to its GitHub repository (automated testing). *mgikit* assigns sample reads exactly the same as splitBarcode output (Tested on DS03 and DS04 in Table 2). We noticed that the order of the reads in splitBarcode’s output is different to the order of the same reads in the input files. *mgikit* maintains the exact read order as they appear in the input files. For additional testing, we used bcl2fastq to convert a Miseq Illumina dataset of 27 207 897 reads into fastq format. We wrote a script to extract the read barcode from the read header and attach it to the read sequence to simulate the output of MGI sequencers. We demultiplexed the formatted dataset with mgikit, and the original dataset with bcl2fastq and both tools found identical matches for all the samples.

**Table 2.** Evaluation of performance time (in minutes) on different datasets.^a^

Dataset	Reads	Samples	Length (bp)		Size (GB)		PE-time		SE-time	
			R1	R2	R1	R2	MK	SB	MK	SB
DS01	298303014	102	300	320	76	85	71.5	84.6	37.2	45.5
DS02	494667136	39	148	172	65	75	61.5	84	31.8	43.1
DS03	506600595	29	100	124	46	55	43.5	65.5	23.2	35
DS04	274567350	5	28	70	8.5	19	13	23.3	11.9	16.5
DS05	500612381	64	50	8	22	5.5	12	31		

a DS01 and DS04 are 10 bp dual index, DS02 and DS3 are 8 bp dual index, and DS05 is 8 bp single index. MK is *mgikit* while SB is splitBarcode. PE is paired-end, while SE is single-end. In the case of the single end, the R2 file of the dataset is used alone for demultiplexing. The size is in Giga Byte for gzip-compressed files. No demultiplexing was done for DS05 in the single-end mode as the R2 contains the index only without sequences. The underlined results indicate the faster tool.

Furthermore, we validated the output of *mgikit* against three other demultiplexers [deML (Renaud *et al.* 2015), Bayexer (Yi *et al.* 2015), and Axe (Murray and Borevitz 2018)] using the dataset described in Renaud *et al.* (2015) and available at https://bioinf.eva.mpg.de/deml/ by comparing the demultiplexed reads of the control sample to the reads aligned to the control region. The results show that *mgikit* and Axe obtain identical results as both tools demultiplex samples based on the hamming distance calculated from the mismatches. *mgikit* and Axe are more conservative in demultiplexing and both assign fewer reads to samples compared to deML and Bayexer. This was reflected in *mgikit* and Axe having the highest precision reaching 97.6% while deML and Bayexer had the highest sensitivity reaching 95.7% and 98.4%, respectively. Details on this analysis and its results are available in the Supplementary Materials.

With regards to performance time evaluation, we assessed *mgikit* (V0.1.3) against splitBarcode (V2.0.0) as they are the only tools that can be used for the same datasets in real scenarios and without reformatting datasets. *mgikit* was between 1.2 and 2.6 times faster than splitBarcode for all tested datasets as shown in Table 2. Tests were done using a single thread on an Intel 2.80 GHz machine with 32 GB RAM and a Linux system. During this evaluation, the peak of memory utilization by *mgikit* was 14 GB compared to 17.8 GB by splitBarcode.

## Conclusion

We provide a tool kit for efficient fastq demultiplexing and quality control reporting that can be utilized for the output of MGI sequencing machines and any other fastq datasets that have index information at the end of the read’s sequence. The tool can also be customized easily to suit different kinds of data.

The evaluation tests showed that *mgikit* obtains identical results to the standard demultiplexers provided by Illumina and MGI. Furthermore, we evaluated hamming distance-based demultiplexers (*mgikit* and Axe) against likelihood-based demultiplexers (deML and Bayexer) that were shown to have more reads assigned to each sample yet with an increased error rate in assigned reads. We believe that the decision on which demultiplexer to use is dependent on several factors such as the quality of the data or what metric to optimize for. Sequencing facilities mainly use standard demultiplexers (mismatch-based approach) before handing data over to users. Reads that are not assigned to any sample after demultiplexing with standard tools can be re-demultiplexed again with other tools to get more reads if desired.

Further developments will focus on improving the performance time through CPU-parallel implementation as well as GPU-based functionality.

## Supplementary Material

btae554_Supplementary_Data

## Data Availability

Datasets used in the evaluation reported in Table 2 can not be shared for privacy reasons. However, we provide a script in the mgikit GitHub repository to simulate datasets with similar features to reproduce this performance evaluation.
