# Supplementary material for: mgikit: demultiplexing toolkit for MGI fastq files
Source: Bioinformatics. 2024 Sep 11;40(9):btae554. doi: 10.1093/bioinformatics/btae554 (PMC11427695; doi:10.1093/bioinformatics/btae554)
Supplement: btae554_Supplementary_Data [file btae554_supplementary_data.pdf]

mgikit: Demultiplexing toolkit for MGI fastq files

Ziad Al Bkhetan and Sen Wang
Supplementary Materials

1 Additional Evaluation

1.1 Testing dataset

For further validation, we downloaded a dataset described at [1] and available at `https://bioinf.eva.mpg.de/deml/`. This dataset contains reads from two regions, one from chromosome 7 of the human genome and the other control region from the PhiX genome. The reads from the control region on the PhiX genome had the indices of the control sample in the sample sheet. The evaluation is to get the demultiplexed reads that are assigned to the control sample and compare them to the reads aligned to the control region in the PhiX genome.

1.2 Read alignment

To find the reads aligned to the control region on PhiX, we trimmed the reads using 'cutadapt' and Illumina universal adapter, and then the reads were aligned to chromosome 7 from the human genome and PhiX genome ('NC_001422.1') using 'bwa' (Version: 0.7.17-r1188) with 'mem' command with its default parameters. Of the 15,245,844 paired reads in the bam file, 19,470,049 reads (considering forward and reverse reads of each pair independently) were aligned to chromosome 7 while 10,270,394 single reads were aligned to the control region in the PhiX genome. Only paired reads with both forward and reverse reads aligned to the PhiX genome are kept for comparison (4,932,249 paired reads).

1.3 Demultiplexing

We compared the results of four tools (deML [1] (Version: 1.1.4), Axe [2] (Version: 0.3.3), Bayexer [3] (Accessed July 2024) and mgikit (Version: v0.1.5)). The dataset was reformatted in different ways to be accepted by Axe and mgikit. This includes appending the indices to the tail of the reversed reads for mgikit demultiplexing, and appending the indices to the beginning of forward and reverse reads for Axe demultiplexing. Demultiplexing was performed using the four demultiplexers and allowing 0, 1, and 2 mismatches for each demultiplexer with an exception to Bayexer as it does not accept such configuration. Bayexer was only executed once with its default parameters. mgikit was applied with disabling format configurations using the following parameters:

```
--disable-illumina --not-mgi --flexible --ignore-undetermined
```

Axe was executed using the following parameters:

```
-z 1 -vv -c
```

deML and Bayexer were executed with their default parameters.

1.4 Accuracy measures

We compared the reads assigned to the control sample (during demultiplexing) to the reads aligned to the control region on the PhiX genome. We calculated the confusion matrix as follows:

1. **True positive (TP):** the number of reads assigned to the control sample during demultiplexing and aligned to the control region during read alignment.
2. **False positive (FP):** the number of reads that are assigned to the control sample during demultiplexing but did not align to the control region during read alignment.
3. **False negative (FN):** the number of reads that were not assigned to the control sample during demultiplexing but were aligned to the control region during read alignment.

Table 1: Evaluation measures for demultiplexing with respect to the reads aligned to the control region on the PhiX genome. No results for Axe when allowing two mismatches as two mismatches cause conflicts in the sample sheet indices and Axe does not allow that. mgikit reports these reads as ambiguous, in other words, it does not assign them to any specific sample. deML assigns them to a sample with the maximum estimated likelihood. Underlined results represent the best-performing tool according to the evaluation metric in the column header. These results are according to the comparison with the reads aligned to the control region (4,932,249 reads).

Tool	Mismatches	Demultipexed reads	FN	FP	TP	Precision	Sensitivity
**mgikit**	0	4547972	491694	**107417**	4440555	**97.6** %	90.0 %
**Axe**	0	4547972	491694	**107417**	4440555	**97.6** %	90.0 %
**deML**	0	4834699	238412	140862	4693837	97.1 %	95.2 %
**mgikit**	1	4786599	281380	135730	4650869	97.2 %	94.3 %
**Axe**	1	4786599	281380	135730	4650869	97.2 %	94.3 %
**deML**	1	4870170	212297	150218	4719952	96.9 %	95.7 %
**mgikit**	2	4881121	213988	162860	4718261	96.7 %	95.7 %
**deML**	2	4869937	212655	150343	4719594	96.9 %	95.7 %
**Bayexer**	-	5044585	**77642**	189978	**4854607**	96.2 %	**98.4** %

We calculated precision as the percentage of reads assigned to the control sample and aligned to the control region of all reads assigned to the control sample. We calculated the sensitivity as the percentage of reads assigned to the control sample and aligned to the control region of all reads aligned to the control region.

1.5 Results

Table 1 shows that the four demultiplexers achieved accurate demultiplexing with precision and sensitivity greater than 90% when increasing the allowed mismatches from 0 to 2. mgikit and Axe were the most conservative tools which is expected with mismatch-based demultiplexers. This led to them achieving the highest precision. Bayexer and deML demultiplexed correctly the largest proportion of reads aligned to the control region with a sensitivity of 98.4% and 95.6%, respectively. We observe that at 2 allowed mismatches, mgikit and Axe demultiplexed reads are more than deML, which is not the behaviour for fewer mismatches (0 and 1). We believe that this is related to the way these tools use the mismatches in their algorithms. Both mgikit and Axe consider the allowed mismatches parameter for each index (similar to the standard demultiplexers such as bcl2fastq and splitBarcode) while deML builds a prefix tree using both indices for a dual-index run and therefore considers the allowed mismatches for both indices. For example, for a dual-index run and 2 allowed mismatches, mgikit and Axe will allow 2 mismatches for each index (4 mismatches for both indices) while deML will only allow 2 mismatches for both indices.

mgikit did the demultiplexing of this dataset in 114 seconds, compared to 232, 668, and 1151 seconds for Axe, deML, and Bayexer (processing uncompressed datasets) respectively. This test was done on a Linux system on Intel(R) Xeon(R) Platinum 8268 CPU @ 2.90GHz machine with 24GB RAM and one CPU.

References

[1] Gabriel Renaud, Udo Stenzel, Tomislav Maricic, Victor Wiebe, and Janet Kelso. deml: robust demultiplexing of illumina sequences using a likelihood-based approach. *Bioinformatics*, 31(5):770–772, 2015.

[2] Kevin D Murray and Justin O Borevitz. Axe: rapid, competitive sequence read demultiplexing using a trie. *Bioinformatics*, 34(22):3924–3925, 2018.

[3] Haisi Yi, Zhe Li, Tao Li, and Jindong Zhao. Bayexer: an accurate and fast bayesian demultiplexer for illumina sequences. *Bioinformatics*, 31(24):4000–4002, 2015.
